# Supplementary material for: Angiography with optical coherence tomography as a biomarker in multiple sclerosis
Source: PLoS One. 2020 Dec 8;15(12):e0243236. doi: 10.1371/journal.pone.0243236 (PMC7723290; doi:10.1371/journal.pone.0243236)
Supplement: Attachment — Submitted filename: R1 review comments.docx [file pone.0243236.s003.docx]

Thank you the effort the author team made to address these comments and providing the datasheet. I studied the revision and datasheet with great interest in understanding the study and conclusion.

There is a **major flaw** on the dataset and analysis, which led to a wrong conclusion. After having a look on the datasheet in the supporting document, I realize that the significant differences of Nasal macula, superior macula and interior macula were due to outliers in the control group. Please see the plots of Superior VD and Nasal VD below. These plots clearly indicated the significance was due to these outliers acquired in a period of time in the control series. This forms a significant problem which led to the wrong conclusion. Without these outliers, statistic analysis showed no significant differences between groups in any sectors. The authors need to report these outliers and include the scatter plots. The authors will also need to report the statistic analysis without these outliers between groups and draw the conclusion. To this reviewer, the data did not differentiate the MS from normal subjects from these scatter plots.

Based on my experience with OCTA, these outliers were due to low quality of images and background noise. The quality control is a major concern on the dataset. The authors will need to examine each case and report the quality scores if these outliers will still be included.

In addition, MSQoL and other measurements were recorded in only 20 patients with MS in Table 7. This represented only about 20% of the patients. The authors failed to mention the missing data and give the wrong impression that all data from all MS patients (n = 92) are obtained.

Furthermore, as suggested in my previous comment, the authors did not cite these recent papers for some unclear reasons (PM31094280 and PM31926161). The authors need to compare their data and conclusion with these recent papers.
