# Supplementary material for: Angiography with optical coherence tomography as a biomarker in multiple sclerosis
Source: PLoS One. 2020 Dec 8;15(12):e0243236. doi: 10.1371/journal.pone.0243236 (PMC7723290; doi:10.1371/journal.pone.0243236)
Supplement: Attachment — Submitted filename: Response to Reviewer.docx [file pone.0243236.s004.docx]

**Thank you the effort the author team made to address these comments and providing the datasheet. I studied the revision and datasheet with great interest in understanding the study and conclusion.**

**There is a major flaw on the dataset and analysis, which led to a wrong conclusion. After having a look on the datasheet in the supporting document, I realize that the significant differences of Nasal macula, superior macula and interior macula were due to outliers in the control group. Please see the plots of Superior VD and Nasal VD below. These plots clearly indicated the significance was due to these outliers acquired in a period of time in the control series. This forms a significant problem which led to the wrong conclusion. Without these outliers, statistic analysis showed no significant differences between groups in any sectors. The authors need to report these outliers and include the scatter plots. The authors will also need to report the statistic analysis without these outliers between groups and draw the conclusion. To this reviewer, the data did not differentiate the MS from normal subjects from these scatter plots.**

**Based on my experience with OCTA, these outliers were due to low quality of images and background noise. The quality control is a major concern on the dataset. The authors will need to examine each case and report the quality scores if these outliers will still be included.**

- Thank you for your effort to thoroughly review our study. We are grateful for your great involvement with this work. All measurements were taken by a single observer, and only images with signal strength index (SSI) and analyzed images with quality score above 50 and 40 respectively were included and also images with movement artefacts were excluded from the analysis.

There is some controversy in the removal or inclusion of outlier data. In our case, delete the most extreme data modifies the content of the article and we want to remain faithful to our study, because this data meets the inclusion and exclusion criteria of quality scores of our study in addition to the fact that it has already been accepted by the two previous reviewers. It is not intended to hide information since these data can be seen reflected in the box diagram of figure 2. However, we are willing, if you consider it, to add a following column to our results with the significance that results from eliminating the data outliers. Eliminating these data, the macular upper quadrant continues to have significant differences between MS patients and healthy controls.

**In addition, MSQoL and other measurements were recorded in only 20 patients with MS in Table 7. This represented only about 20% of the patients. The authors failed to mention the missing data and give the wrong impression that all data from all MS patients (n = 92) are obtained.**

- We have added a clarifying phrase to add the number of patients who did the quality of life questionnaire MSQoL-54 and also, we add this in limitations of the study.
  - Line 203: MSQoL-54 was completed by 20 MS patients..
  - Line 348: The number of patients who answered MSQoL-54 was small compared to the total sample

**Furthermore, as suggested in my previous comment, the authors did not cite these recent papers for some unclear reasons (PM31094280 and PM31926161). The authors need to compare their data and conclusion with these recent papers.**

- We have added these recent papers in our discussion
  - Line 299: In this study, a significant decrease in VD was observed in the superior, nasal and inferior macular areas in patients suffering from MS with Triton Plus OCTA. Our results support previous research, such as Lanzillo R et al [12] (with Octovue OCTA device) and Olwen C Murphy et al [29] (Spectralis OCTA), both showing a loss of VD in SVP macular ETDRS in MS patients. Only one study evidenced an increase in VD in MS patients [30], which was realized with Angioplex Zeiss device.

Thank you for your reviewers to improve quality of this study.

We hope that these changes work out your doubts.
