# Supplementary material for: Angiography with optical coherence tomography as a biomarker in multiple sclerosis
Source: PLoS One. 2020 Dec 8;15(12):e0243236. doi: 10.1371/journal.pone.0243236 (PMC7723290; doi:10.1371/journal.pone.0243236)
Supplement: Attachment — Submitted filename: Responses to reviewer.docx [file pone.0243236.s005.docx]

Thank you again for your revision. We review and explain your suggestions one by one below.

We would like the reviewers to clarify where the paragraphs considered incorrect in English redaction are. This paper was reviewed by an English native editor but we can edit the manuscript again for a better understanding.

1. As the authors indicated in their responses to the issue of outliers, this reviewer believes that it is necessary to include new columns (mean ± SD and P value) of the control group and P values in comparison of the MS group in Tables 3, 4, 5 and 6.

As the reviewer proposed here, we have included new data in tables 3, 4, 5 and 6: mean ± SD value in control and EM groups without outliers and P value with these data.

2. The authors indicated that the macular upper quadrant continues to have significant differences between MS and controls after eliminating the data outliers. However, the t-test with two tails yielded P value as 0.495, a marginal significant level. Recommend to further discuss.

In the superior macular quadrant, p value is 0.047 after removing outliers.

Line 219: Superior macular area remains minimally significantly low vascular density in EM groups despite removing the outlier (p =0.047).

Line 312: More precisely, VD decrease remains significant when we remove outliers.

Line 320: In light of these results, it may be hypothesized that a relationship exists between the decrease in the temporal and superior peripapillary RNFL and vasculature that nourishes this layer, especially in superior macular area that shows a marked decrease in our results. Feucht et al [33] in his study shows this with a high correlation between loss retinal vasculature and thinning retinal layers.

3. In addition, to better interpret the data, averaged vascular density of the macular area (averaged from 4 quadrants) needs to be included in these tables, which will conclude whether the vascular density altered in the macular area in patients with MS. This will also facilitate the comparison of their work to the previously published work.

A row was added with average vascular density of the 4 quadrants of the macular area in tables 3, 4, 5 and 6 for a better interpretation the data.

4. Recommend to include a scatterplot (subject sequential ID vs measurements) in addition to Figure 2. Figure 2 does not show the sequence of data collection. The authors will also need to discuss the possible reasons why the outliers happened in the data’s early collection

A scatterplot of average macular vascular density was added as Figure 2. Studies like Orlov et al, show that there is a variability in retinal microvasculature due to age. In our study variability in values of vascular density is present but we consider this variability representative of a normal population sample and only images of high quality were used in our study.

Line 210: It is necessary to consider that retinal vasculature has some anatomical variations sensitive to axial length, refraction, age… (26), which causes variability in vascular density values. Figure 2 show VD values in control group and EM group related with age. (Fig 2)

**Fig 2. Scatterplot graphic of vascular density and age in control group and MS group.**

26- Orlov NV, Coletta C, van Asten F, Qian Y, Ding J, AlGhatrif M, et al. Age-related changes of the retinal microvasculature. PLoS One. 2019 May 2;14(5): e0215916.
